# Antimicrobial Susceptibility Breakpoints and First-Step *parC* Mutations in *Streptococcus pneumoniae*: Redefining Fluoroquinolone Resistance

**DOI:** 10.3201/eid0907.020589

**Published:** 2003-07

**Authors:** Sue Lim, Darrin Bast, Allison McGeer, Joyce de Azavedo, Donald E. Low

**Affiliations:** * Toronto Medical Laboratories/Mount Sinai Hospital, Toronto, Ontario, Canada; † University of Toronto, Toronto, Ontario, Canada

**Keywords:** fluoroquinolone resistance, levofloxacin resistance, antimicrobial susceptibility testing, topoisomerase, research

## Abstract

Clinical antimicrobial susceptibility breakpoints are used to predict the clinical outcome of antimicrobial treatment. In contrast, microbiologic breakpoints are used to identify isolates that may be categorized as susceptible when applying clinical breakpoints but harbor resistance mechanisms that result in their reduced susceptibility to the agent being tested. Currently, the National Committee for Clinical Laboratory Standards (NCCLS) guidelines utilize clinical breakpoints to characterize the activity of the fluoroquinolones against *Streptococcus pneumoniae*. To determine whether levofloxacin breakpoints can identify isolates that harbor recognized resistance mechanisms, we examined 115 *S. pneumoniae* isolates with a levofloxacin MIC of >2 μg/mL for first-step *parC* mutations. A total of 48 (59%) of 82 isolates with a levofloxacin MIC of 2 μg/mL, a level considered susceptible by NCCLS criteria, had a first-step mutation in *parC*. Whether surveillance programs that use levofloxacin data can effectively detect emerging resistance and whether fluoroquinolones can effectively treat infections caused by such isolates should be evaluated.

The emergence of *Streptococcus pneumoniae* resistance to β-lactam and macrolide antimicrobial agents has led to recommendations that fluoroquinolones with increased activity against *S. pneumoniae*, such as levofloxacin, moxifloxacin, and gatifloxacin, be used to treat patients at risk for infection caused by such multidrug-resistant strains (*1*–*6*). Fluoroquinolone resistance in *S. pneumoniae* is primarily due to mutations in the genes encoding the target topoisomerase enzymes, namely *parC*, which encodes the A subunit of DNA topoisomerase IV, and *gyrA*, which encodes the A subunit of DNA gyrase (*7*). Mutations in *parE* and *gyrB* have been reported, but to a lesser extent (*8*–*10*). Most pneumococcal isolates with reduced susceptibilities to fluoroquinolones have amino acid substitutions in either ParC alone or ParC and GyrA (*11*–*14*). Resistance can also be mediated by active efflux (*15*), although the role of efflux in contributing to resistance by the newer fluoroquinolones is unclear (*16*).

The MIC of an antimicrobial agent is a value that has been used to determine breakpoints that predict the probability of clinical success, detect resistant populations, or both (*17*). Clinical breakpoints are dependent on the antimicrobial activity and pharmacology of the drug; such breakpoints are ascertained with the goals of eradicating the infection and ultimately achieving clinical success with the antimicrobial agent. In contrast, microbiologic breakpoints are established to identify isolates that may be categorized as susceptible when applying clinical breakpoints but harbor resistance mechanisms that result in their reduced susceptibility to the agent being tested. These microbiologic breakpoints are therefore useful in monitoring the emergence of resistance. The current National Committee for Clinical Laboratory Standards (NCCLS) guidelines make no distinction between these two interpretations of MIC, with clinical breakpoints used to characterize most antimicrobial agents, including the fluoroquinolones.

Levofloxacin has been used as a surrogate marker to predict fluoroquinolone susceptibility in clinical laboratories and surveillance studies (*18*). To establish whether current levofloxacin breakpoints are also able to function as microbiologic breakpoints, we determined the percentage of *S. pneumoniae* isolates with first-step *parC* mutations that would go undetected by using the current NCCLS breakpoints for levofloxacin (*19*).

## Materials and Methods.

A total of 6,076 clinical isolates of *S. pneumoniae* were collected as part of a 1993–1998 surveillance program throughout Canada. All isolates were identified as *S. pneumoniae* by standard methods. The isolates were frozen at –70°C, thawed, subcultured onto blood agar, and incubated at 37°C in 5% CO_2_ for 24 h twice before testing. In vitro susceptibility testing was performed by broth microdilution, according to NCCLS guidelines (*20*,*21*). Susceptibility interpretive criteria used were those published in the NCCLS M100-S12 document (*19*). The nonsusceptible category was defined as those isolates with MICs of fluoroquinolines in the intermediate and resistant category. The *parC* gene of 115 isolates with a levofloxacin MIC >2 μg/mL (82 = MIC 2 μg/mL; 8 = MIC 4 μg/mL; 10 = MIC 8 μg/mL; and 15 = MIC ≥16 μg/mL) was amplified by polymerase chain reaction (PCR), and the nucleotide sequence determined as previously described (*9*). All isolates (n=33) with a levofloxacin MIC of ≥4 μg/mL, and a random sample of 29 isolates with a levofloxacin MIC of 2 μg/mL were examined for *gyrA* mutations. For comparative purposes, the *parC* gene of 14 isolates with a ciprofloxacin MIC of 2 μg/mL, regardless of their levofloxacin MIC, was amplified and sequenced. Although numerous single mutational events occur in *parC*, the focus of this investigation was on amino acid substitutions for Ser-79 or Asp-83, because previous studies have consistently demonstrated that mutations at either of these positions are associated with decreased susceptibility (*9*,*14*).

Crude cell lysates were used as DNA templates for PCR. After the cells were grown overnight growth on Columbia nutrient agar and supplemented with 5% sheep blood, a loopful of growth was suspended in 100 μL of lysis buffer (100 mM NaCl, 10 mM Tris-HCl [pH 8.3], 1 mM EDTA, 1% Triton X-100) and boiled for 10 min. Ten microliters of the supernatant was used as the template in a 50-μL reaction volume. The quinolone-resistance–determining regions of *parC* and *gyrA* were amplified by PCR. Primers used were based on published sequences (*7*,*8*), and amplification products were purified with either the QIAquick PCR purification kit (Qiagen Inc., Mississauga, Ontario, Canada) or the Concert Rapid PCR purification kit (Life Technologies, Burlington, Ontario, Canada).

DNA sequencing was performed by ABI prism Big Dye terminator cycle sequencing with the ABI 377 automated sequencer (PE Applied Biosystems, Mississauga, Ontario, Canada). Nucleotide and amino acid sequence comparisons were performed by the multiple-alignment sequence function of Vector NTI Suite software (InforMax Inc., Bethesda, MD). The GenBank accession numbers for the wild-type sequences used for comparison purposes were Z67739 for *parC* and *parE* (*22*), AB010387 for *gyrA*, and Z67740 for *gyrB* (*23*).

Isolates were examined for active efflux by agar dilution on Mueller-Hinton agar containing 5% sheep blood in the presence of ciprofloxacin with or without 10 mg/mL of reserpine (Sigma Chemical Co., St. Louis, MO) (*24*). Strains for which a fourfold or greater decrease in the MIC of ciprofloxacin existed n the presence of reserpine were considered in this study to be positive for reserpine-inhibited efflux. *S. pneumoniae* strain P121/1N27 and clinical isolate BSP 823 were used as quality control strains, the latter of which demonstrated a 16-fold decrease in the ciprofloxacin MIC in the presence of reserpine (*9*).

## Results

Of the 115 *S. pneumoniae* isolates with a levofloxacin MIC >2 μg/mL, 78 (69%) had an amino acid substitution in ParC (Ser-79 or Asp-83) (Table 1). Mutations in *gyrA* were not found in any of the randomly selected isolates with a levofloxacin MIC of 2 μg/mL, but were present in three (38%) of eight isolates with a levofloxacin MIC of 4 μg/mL and in all isolates with a levofloxacin MIC ≥ 8 μg/mL (Table 2). The specific ParC amino acid substitutions of the isolates and their corresponding levofloxacin MICs are shown in Table 1. The most common substitution was Ser-79 to Phe, accounting for 60% of all observed amino acid substitutions. The prevalence of first-step ParC amino acid substitutions among all strains according to their levofloxacin and ciprofloxacin MICs is shown in Table 3. Using the current MIC interpretive standards for levofloxacin, 48 (59%) of 82 of isolates with a first-step mutation fall in the susceptibility category of levofloxacin (MIC<4 μg/mL). In comparison, 4 (29%) of 14 randomly chosen isolates with a ciprofloxacin MIC of 2 μg/mL harbored a first-step mutation.

**Table 1:** ParC amino acid substitutions found in 115 *Streptococcus pneumoniae* isolates with levofloxacin MICs >2 μg/mL and corresponding levofloxacin MICs

ParC amino acid substitution	No. isolates inhibited by levofloxacin MIC (µg/mL) of					Total no. of strains
	2	4	8	16	≥32	
Ser79→Phe	28	4	3	9	3	47
Ser79→Tyr	7	1	3	2	1	14
Ser79→Ala	1	0	0	0	0	1
Asp83→Asn	7	0	3	0	0	10
Asp83→Gly	1	0	0	0	0	1
Asp83→Tyr	3	0	0	0	0	3
Asp83→Val	1	0	0	0	0	1
Asp83→Ala	0	0	1	0	0	1
No. isolates/total with amino acid substitutions	48/82 (59%)	5/8^a^ (63%)	10/10	11/11	4/4	78/115 (69%)

^a^One isolate with no ParC amino acid substitution found to have active efflux; two isolates had ParC amino acid substitutions at sites other than Ser79 or Asp83.

**Table 2:** Number of isolates with ParC and GyrA amino acid substitutions and their corresponding levofloxacin MICs

MIC (μg/mL)	No. strains with amino acid substitutions in	
	ParC (%)	ParC and GyrA (%)
2	48/82 (59)	0/29^a^ (0)
4	5/8 (63)	3/8 (38)
8	0/10 (0)	10/10 (100)
≥16	0/15 (0)	15/15 (100)

^a^29/82 isolates were randomly examined for GyrA mutations.

**Table 3:** The prevalence of ParC amino acid substitutions among all strains according to their levofloxacin and ciprofloxacin MICs

MIC (μg/mL)	No. strains with ParC amino acid substitution at 79 or 83	
	Ciprofloxacin (%)	Levofloxacin (%)
2	4/14 (29)	48/82 (59)
4	24/37 (65)	5/8 (63)
8	11/12 (92)	10/10 (100)
>8	22/22 (100)	15/15 (100)
Total	62/87 (71)	78/115 (69)

Thirty-three isolates were nonsusceptible to levofloxacin (MIC>4 μg/mL); for 25, which harbored both ParC and GyrA amino acid substitutions, the levofloxacin MIC was ≥8 μg/mL (Table 3). For eight isolates, the levofloxacin MIC was 4 μg/mL; three (38%) of those isolates had a substitution in GyrA (Ser-81-Phe) as well as a substitution in ParC (Ser-79-Phe, Asp-78-Asn and Ala-115-Pro) (Table 4). In addition, two of the eight (25%) isolates had no substitution in GyrA, but were considered positive for reserpine-inhibited efflux, while three isolates had a Ser-79-Phe amino acid substitution in ParC. No mutations were found in either *parE* or *gyrB* in the isolates with a levofloxacin MIC of 4 μg/mL.

**Table 4:** Characterization of *Streptococcus pneumoniae* isolates with levofloxacin MIC 4 μg/mL^a^

Isolate no.	Amino acid substitution		Change in MIC with inhibition of efflux
	In ParC	In GyrA	
1	Ser79→Phe	None	8-fold
2	Ser79→Phe	None	No effect
3	Asp78→Asn	Ser81→Phe	No effect
4	Ala115→Pro	Ser81→Phe	No effect
5	Ser79→Phe	None	No effect
6	Ser79→Phe	None	No effect
7	None	None	4-fold
8	Ser79→Phe	Ser81→Phe	No effect

^a^*parE* and *gyrB* sequencing was performed on all isolates, but no mutations were found in the quinolone-resistance–determining region.

## Discussion

Before the development of fluoroquinolones such as levofloxacin, ofloxacin was used to determine trends of pneumococcal fluoroquinolone resistance in the United States (*25*). By using this system, an increase of ofloxacin-nonsusceptible isolates from 2.6% in 1995 to 3.8% in 1997 was reported. However, the significance of such an increase was questioned, since ofloxacin-resistant strains could be observed with only a single topoisomerase mutation, whereas for fluoroquinolones such as levofloxacin, multiple mutations are required for a strain to be classified as resistant according to NCCLS breakpoints (*25*–*27*). As a consequence, ofloxacin was replaced by levofloxacin in 1998 as a marker for fluoroquinolone nonsusceptibility, and not surprisingly, given levofloxacin’s increased activity against *S. pneumoniae*, fluoroquinolone resistance rates were only 0.2% in 1998 and 1999 (*25*).

Since effective surveillance depends upon the ability to detect the emergence of resistance, the prevalence of pneumococci that harbor resistance mechanisms to the fluoroquinolones may not be accurately represented if surveillance systems that rely on levofloxacin MIC data are used (*25*,*28*–*34*). We found that 59% of isolates with a levofloxacin MIC of 2 μg/mL, a level considered susceptible according to NCCLS criteria, had a first-step mutation in *parC*. Similarly, Davies et al. (*12*) found that of 14 strains for which levofloxacin MICs were 2 μg/mL, 10 (71%) had a *parC* mutation. Therefore, if the goal of surveillance is to detect emerging problems, then by extension, the detection of first-step mutations is also important and the use of current NCCLS breakpoints to estimate fluoroquinolone resistance is clearly inadequate. Apart from DNA sequencing, currently no accurate test can reliably identify isolates with first-step mutations (*35*). Although decreasing levofloxacin breakpoints has been proposed as a solution to this problem, we found that 8 (25%) of 32 strains for which the levofloxacin MIC was 1 μg/mL already had first-step mutations (data not shown). Similarly, the replacement of levofloxacin as a surveillance indicator by another fluoroquinolone has also been suggested. However, use of ciprofloxacin does not fare significantly better, with 4 (29%) of 14 isolates in the susceptible category (MIC of 4 μg/mL to define nonsusceptible isolates) harboring first-step mutations.

In addition to causing an underestimation of the emergence of fluoroquinolone resistance, the use of clinical breakpoints has therapeutic implications, as supported by recent reports of treatment failure when a fluoroquinolone was used to treat an infection caused by a strain of pneumococci with a first-step mutations (*36*,*37*). Clearly, a first-step mutation is necessary for the development of subsequent mutations, the latter of which result in MICs that fall within the nonsusceptible category. However, studies have shown that upon acquisition of a first-step mutation, the likelihood of developing a subsequent mutation is enhanced in comparison to the development of the first-step mutation itself (*38*–*40*). Studies are required to determine whether isolates with one or more mutations in genes encoding ParC, GyrA, or both, can still be effectively treated with a fluoroquinolone when that fluoroquinolone is found to be susceptible by using current clinical breakpoints. Recognizing the presence of underlying mutations may be especially important when using these agents to treat patients with large biomass infections such as pneumococcal pneumonia.

Lastly, the acquisition of a second-step mutation appears more likely than not to raise the MIC to >8 μg/mL and not to 4 μg/mL as would be expected. Isolates with a levofloxacin MIC of 4 μg/mL represented 0.1% of the total number of isolates in our study, which is notable, considering that a levofloxacin MIC of 4 μg/mL is currently used to define nonsusceptibility. Furthermore, these isolates were for the most part either genotypically or phenotypically distinct from other isolates characterized (Table 4); two had efflux mechanisms, one singly and the other concurrent with a ParC amino acid substitution, and two had unusual substitutions in ParC (Asp78→Asn and Ala115→Pro). The importance of this latter finding remains to be determined.

In summary, levofloxacin susceptibility testing that uses current MIC clinical breakpoints does not identify most *S. pneumoniae* isolates with only first-step *parC* mutations. This finding may not only have implications for the ability of surveillance programs to detect emerging resistance, but therapeutic implications as well.
